# Harnessing spectrophotometry resolution power for determining ternary mixture for respiratory disorders treatment in their pharmaceutical formulation

**DOI:** 10.1371/journal.pone.0311121

**Published:** 2024-10-07

**Authors:** Huda Salem AlSalem, Faisal K. Algethami, Maimana A. Magdy, Nourudin W. Ali, Hala E. Zaazaa, Mohamed Abdelkawy, Mohammed Gamal, Maha M. Abdelrahman

**Affiliations:** 1 Department of Chemistry, College of Science, Princess Nourah Bint Abdulrahman University, Riyadh, Saudi Arabia; 2 Department of Chemistry, College of Science, Imam Mohammad Ibn Saud Islamic University (IMSIU), Riyadh, Saudi Arabia; 3 Faculty of Pharmacy, Pharmaceutical Analytical Chemistry Department, Beni-Suef University, Alshaheed Shehata Ahmad Hegazy St., Beni-Suef, Egypt; 4 Faculty of Pharmacy, Pharmaceutical Analytical Chemistry Department, Cairo University, Cairo, Egypt; Siksha O Anusandhan University School of Pharmaceutical Sciences, INDIA

## Abstract

A ternary mixture incorporating Hydroxyzine hydrochloride (HYX), Ephedrine hydrochloride (EPH) and Theophylline (THP) frequently prescribed for the treatment of respiratory diseases. Herein, two spectrophotometric methods are designated and applied to resolve these three components in their mixture. Method A is ratio-subtraction combined with derivative spectrophotometry, where THP can be determined directly at its λ_max_ 271 nm (neither HYX or EPH interfere), then for determination of HYX and EPH, the ternary mixture was divided by 22 μg/mL of THP and after subtraction of the plateau region, HYX can be determined directly at its λ_max_ 234.2 nm (absence of EPH intervention). Finally, the third derivative (^3^D) spectrophotometric approach was utilized to estimate EPH by detecting the peak amplitude at 222 nm with Δλ = 4 and a scaling factor 100. Principal Component Regression (PCR) and Partial Least Squares (PLS), two multivariate calibration approaches, were applied effectively in Method B. This method effectively quantified the mixture under investigation by using the absorption spectra obtained from suitable solutions of the three components in the 210–230 nm region. The calibration models were evaluated using cross-validation with PCR and PLS, producing statistical characteristics that demonstrate the effectiveness of the calibration models. Synthetic and pharmaceutical preparations were also used to conduct external validation. In pharmaceutical formulation, these methods were successfully applied to analyze HYX, EPH, and THP without overlap from formulation’s excipients. Moreover, the study’s findings were statistically contrasted with those of earlier reported HPLC method. Appraisal approaches were used to determine whether the new spectrophotometric methods had an adverse environmental impact involving the Green Analytical Procedure Index (GAPI) and the AGREE (Analytical Greenness). These evaluations delivered information about the methods’ eco-friendliness and sustainability, proving that they are in line with ecologically attributed practices. Furthermore, the Blue Applicability Grade Index (BAGI) was utilized to identify and verify the feasibility and practicality of the suggested approaches.

## 1. Introduction

**Fig 1:**
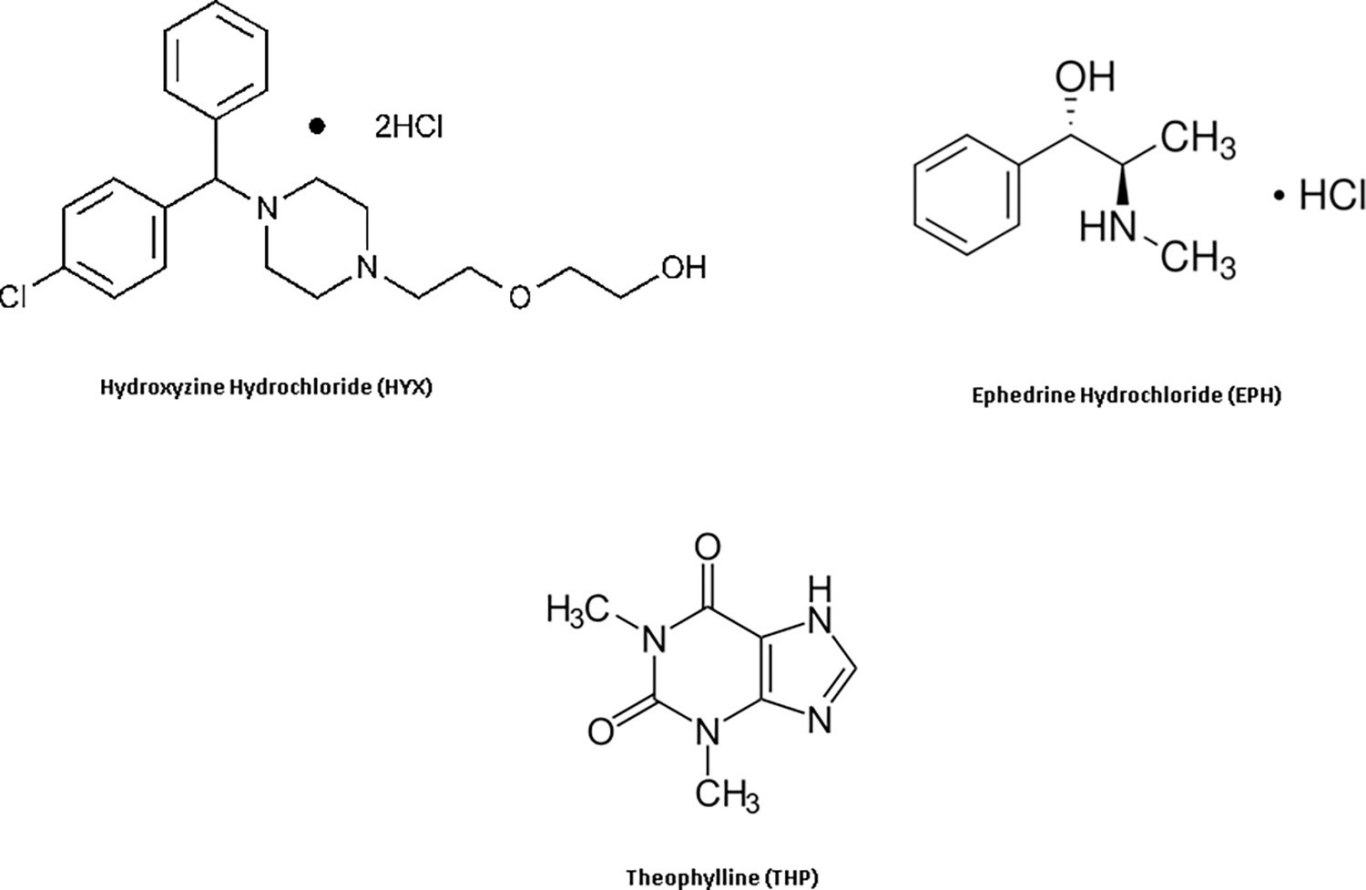
Chemical structure of Hydroxyzine hydrochloride (HYX), Ephedrine hydrocloride (EPH) and Theophylline (THP).

Hydroxyzine hydrochloride (HYX) chemically identified as (RS)-2-[2-[4-[(4-chlorophenyl)phenylmethyl]piperazin-1-yl]ethoxy] ethanol dihydrochloride [1], HYX is a diphenylmethane and piperazine class first-generation antihistamine **(Fig 1)**. It is stated to have strong anxiolytic and mild anti-obsessive as well as antipsychotic properties [2]. EPH, also known as Ephedrine Hydrochloride, is a chemical compound with the chemical name (1R,2S)-2-(methylamino)-1-phenylpropan-1-ol hydrochloride [1], **Fig 1**. It is classified as a sympathomimetic amine and is commonly used for various medical purposes. EPH is used to treat hypotension related to anesthesia, as well as for decongestant and appetite suppressant purposes [3]. Theophylline (THP), chemically known as 1,3-dimethyl-3,7-dihydro-1H-purine-2,6-dione [1], **Fig 1**. It is used for respiratory diseases such as relaxing bronchial smooth muscle, and asthma [4].

The British Pharmacopeia (BP) [1] and the United States Pharmacopeia (USP) [5] both provide titrimetric methods for quantifying HYX, EPH, and THP, separately, in raw materials [6]. HYX, EPH and THP were determined either alone or in combination with other components by HPLC [7–20], TLC-densitometry [21–26], GC [17,27,28], electrochemical [29–34] and different spectrophotometric methods [17,35–40]. After conducting a literature review, only two HPLC techniques were found for the simultaneous measurement of HYX, EPH, and THP [41,42].

Derivative, ratio-subtraction, and multivariate calibration are crucial techniques in the investigation of mixtures containing several components in the field of UV-VIS molecular absorption spectrophotometry. These methods are useful in overcoming issues such as spectrum overlap and interference, which enables accurate and consistent quantification of individual components within complicated mixes. The use of derivative and ratio-subtraction techniques can improve the spectrum information, allowing for better analyte discrimination and determination. Furthermore, multivariate calibration approaches such as Principal Component Regression (PCR) and Partial Least Squares (PLS) are effective in modeling and forecasting the concentrations of numerous components simultaneously. The combination of derivative and ratio-subtraction approaches, as well as multivariate calibrations, has significantly advanced multi-component analysis in UV-VIS molecular absorption spectrophotometry [43,44].

Two spectrophotometric methods are proposed for detecting HYX, EPH, and THP in their mixed form. The methods demonstrate adequate sensitivity, accuracy, and precision. The approaches’ simplicity and speed are additional advantages. As per the ICH guidelines [45], the proposed methods have been verified.

## 2. Experimental

### 2.1. Instruments

The experiment involved using a UV-1601 PC double-beam UV-visible spectrophotometer (manufactured by SHIMADZU, Japan) was connected to an IBM-compatible PC. The spectrophotometer had a quartz cell with a path length of one centimeter, and version 3.7 of the UV-PC personal spectroscopy software was used to run the experiment. The wavelength-scanning speed was 2800 nm/min, with a spectral bandwidth of 2 nm. MATLAB^®^, version 6.5, PLS-Toolbox 2.0 was used for all data analysis [46].

### 2.2. Materials

#### (a) Pure standard

The Chemical Industries Development (CID) Company in Giza, Egypt provided theophylline and hydrochlorides of hydroxyzine and ephedrine. The HPLC method showed purity values of 100.52, 99.85, and 99.32 for HYX, EPH, and THP, accordingly [41].

#### (b) Pharmaceutical formulation

Bronchaline^®^ tablets (Batch No. 14311W), produced by Chemical Industries Development (CID) Company, Giza, Egypt, are marked to include 10 mg of HYX, 15 mg of EPH, and 120 mg of THP.

#### (c) Solvents and chemicals

During this investigation, analytical-grade chemicals and reagents without any additional purification were worked. HPLC-grade methanol (Sigma-Aldrich Chemie Gmbh, Germany; CHROMASOLV^®^) was handled.

### 2.3. Standard solutions

(a) Stock standard solutions (1 mg/mL in methanol): weigh 0.1 g of HYX, EPH, and THP separately and accurately to three different 100-mL volumetric flasks. Add 50 mL of methanol to each flask and agitate it until the contents are dissolved. Then, fill up the flask to the mark with methanol.

(b) Working standard solutions of HYX, EPH and THP (100 μg/mL): Pour 10 mL of each HYX, EPH, and THP stock solutions into three separate 100-mL volumetric flasks. Add enough methanol to each flask to reach the flask’s capacity mark.

### 2.4. Laboratory prepared mixtures

Prepare different mixtures with various proportions of the investigated drugs HYX, EPH and THP comprising the commercial formulation ratio using their corresponding working solutions.

## 3. Methodology

### 3.1. Spectral characteristics of hydroxyzine hydrochloride, ephedrine hydrochloride and theophylline

The absorption spectra of 10 μg/mL of HYX, 15 μg/mL of EPH and 10 μg/mL of THP, were recorded using methanol as a blank in the range of 200–400 nm.

### 3.2. Ratio-subtraction combined with derivative spectrophotometric method

#### 3.2.1. Linearity and construction of calibration curves

To prepare for analysis, precise amounts of HYX, EPH, and THP were measured and placed into separate 10-mL volumetric flasks. The amounts were 20–200 μg, 30–350 μg, and 20–240 μg, accordingly, and each flask was filled with methanol. Between 200 and 400 nm, the zero-order absorption spectra of every group were examined. To determine the concentration of THP, the absorption value at 271 nm was recorded from the stored zero-order absorption spectra. A calibration curve was then created, relating the absorption values at the chosen wavelength to their associated concentrations. Finally, a regression equation was calculated from the calibration curve.

HYX absorption spectra were divided by the standard spectrum of 22 μg/mL of THP to calculate HYX. Using the resulting spectra, peak amplitude at 234.2 nm can be measured. Similarly, to determine EPH, the absorption spectra of EPH were divided by the standard spectrum of 22 μg/mL of THP. Next, ^3^D spectra of EPH solutions were acquired using Δλ = 4 and a scaling factor of 100, with the peak amplitude being detected at 222 nm. Regression equations were derived using calibrated graphs that were constructed to connect the peak amplitudes with the relevant drug concentrations.

#### 3.2.2. Assay of laboratory prepared mixtures

Different mixtures of HYX, EPH, and THP were produced. The absorption value at 271 nm of each mixture’s absorption spectra was then determined to figure out the level of THP using the respective regression algorithm.

The quantity of HYX in each mixture could be calculated by dividing the absorption spectra by the standard spectrum of 22 g/mL THP. The next step involved deriving the subtraction spectrum by subtracting amplitude magnitude in the plateau region for each of the spectra-divided obtained at 271 nm. Subsequently, the absorbance value of the subtraction spectrum was acquired at 234.2 nm, and the quantity of HYX in each mixture was found using its regression formula.

After dividing the produced mixture by 22 μg/mL of THP, the subtraction spectrum was generated. Then, using Δλ = 4 and scaling factor 100, ^3^D spectra were created, from which the concentration of EPH at 222 nm could be revealed from its regression equation.

### 3.3. Multivariate calibration techniques

#### 3.3.1. Construction of the training set

A calibration set of 25 samples in methanol was generated using a multilevel multifactor design [33]. The design employed a five-level, three-factor calibration design.

According to **Table 1**, the values fell within the calibration range of 2–10 μg/mL for HYX, 3–15 μg/mL for EPH, and 2–18 μg/mL for THP. These samples’ digital UV absorption spectra were gathered every 0.2 nm between 210 and 230 nm. The PLS-Toolbox program version 6.5 was used to perform the computation. Afterward, the UV-absorption spectra of these mixes were examined using PCR and PLS-1 models.

**Table 1:** The concentration of mixtures of hydroxyzine hydrochloride, ephedrine hydrochloride and theophylline used in the training sets.

Mixture No.	HYX (μg/mL)	EPH (μg/mL)	THP (μg/mL)
1	6	9	10
2	8	15	14
3	10	12	10
4	8	9	18
5	6	15	18
6	10	15	2
7	10	3	14
8	2	12	2
9	8	3	10
10	2	9	14
11	6	12	14
12	8	12	6
13	8	6	2
14	4	3	6
15	2	6	10
16	4	9	2
17	6	3	2
18	2	3	18
19	2	15	6
20	10	6	18
21	4	15	10
22	10	9	6
23	6	6	6
24	4	6	14
25	4	12	18

#### 3.3.2. Picking the ideal number of variables

When performing PCR and PLS calibrations, it is fundamental to select the appropriate number of principal components or factors. To avoid overfitting, the number of variables should be chosen by taking into consideration as much of the experimental data as possible. Various parameters have been developed to determine the optimal number. During the cross-validation process, leaving out one sample at a time was employed [47].

In each calibration sample, the expected and actual amounts of the substances were compared to determine the root mean squares error of cross-validation (RMSECV). Every time we added a additional variable to the model, then computed the RMSECV in the same way. The ideal number of components was visually determined. Mean centering the data improved both PLS and PCR outcomes after constructing the models.

#### 3.3.3. Construction of the validation set

We created seven distinct combinations of HYX, EPH, and THP by importing varying volumes of their respective working standard solutions, as listed in Table 2. We then used the created models to estimate the concentrations of HYX, EPH, and THP for each mixture.

**Table 2:** Determination of hydroxyzine hydrochloride, ephedrine hydrochloride and theophylline in laboratory prepared mixtures by the proposed ratio-subtraction combined with derivative spectrophotometric method.

Mix No.	Concentration (μg/mL)			Recovery ^b^%		
				Ratio-subtraction combined with derivative spectrophotometric method		
	HYX	EPH	THP	HYX	EPH	THP
				at 234.2 nm	at 222 nm	at 271 nm
1	10	10	10	102.60	102.00	103.50
2	3	6	9	101.33	98.00	101.55
3	5	10	15	101.40	101.70	101.60
4	6	10	20	99.17	98.80	99.50
5	5	5	5	101.00	101.00	100.60
6	8	10	14	99.62	102.90	102.28
7^a^	2	3	24	103.00	102.33	101.25
**Mean ±SD**				101.16± 1.408	100.96± 1.857	101.47± 1.257

^**a**^ The ratio of the studied drugs in Bronchaline^®^ tablets.

^**b**^ Average of 3 determination.

### 3.4. Applying to pharmaceutical formulation (Bronchaline^®^ tablets)

Twenty tablets of Bronchaline^®^ were thoroughly combined and powdered well. In a 100-mL volumetric flask, a precisely weighed amount of the powdered tablet containing 120 mg of THP was placed. After adding 75 mL of methanol, the flask was subjected to ultrasound for 30 minutes, filtered, and then filled with methanol. The prepared solution was used to make the stock standard solution for THP (1200 μg/mL), the working standard solution for HYX (100 μg/mL), and the working standard solution for EPH (150 μg/mL). Methanol is used as a diluent to dilute a portion of the solution mentioned above, resulting in a working solution for THP (300 μg/mL). After that, HYX, EPH, and THP were analyzed using the previously described methods, and their concentrations were determined using the related regression formula.

## 4. Results and discussion

### 4.1. Ratio-subtraction combined with derivative spectrophotometric method

Several established spectrophotometric techniques can assess pharmaceutical mixtures without pre-separation procedures. The derivative and ratio-subtraction processes are advantageous for analyzing binary and ternary mixtures [48].

Combining the "ratio subtraction" and "ratio spectra derivative" procedures simplifies spectrophotometric processes, enabling the resolution of ternary combinations [49]. When dealing with ternary mixtures, a combination of different strategies can be used to address the issue more efficiently. By using the ratio-subtraction approach, it’s possible to eliminate any interference caused by other components. Furthermore, the ratio spectrum derivative method can be employed to improve discrimination between components. This merging approach accurately quantifies the different components of a mixture, eliminating the need for pre-separation. It can effectively resolve ternary mixtures using "ratio-subtraction" and "ratio spectra derivative" procedures in spectrophotometric techniques. This method has broad applications in complex mixture analysis, including pharmaceutical analysis, and other fields [50].

HYX, EPH, and THP absorption spectra greatly overlap, hence no spectrophotometric technique has ever been constructed to measure them. As conventional spectrophotometric methods failed to resolve the overlap between the studied components, this manuscript proposes a new spectrophotometric method utilizing ratio subtraction and derivative spectrophotometry.

**Fig 2:**
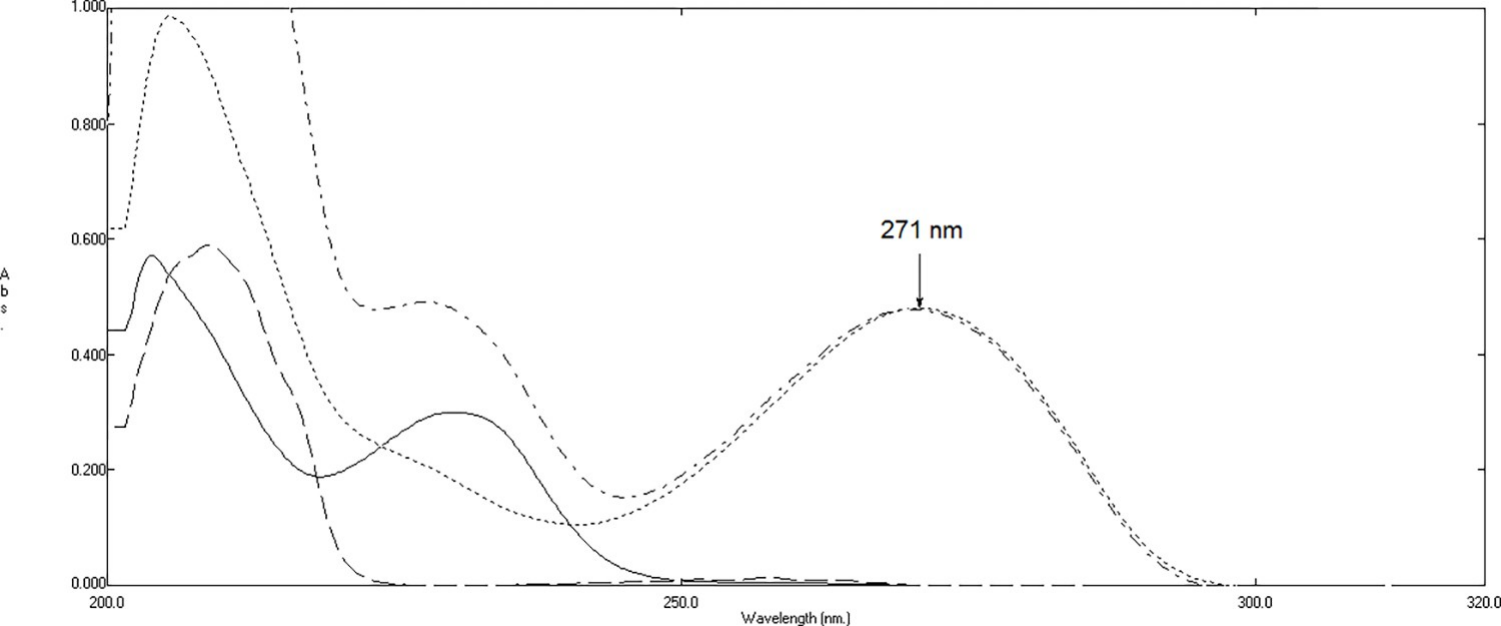
Zero order absorption spectrum of 10 μg/mL of hydroxyzine hydrochloride (―), 15 μg/mL of ephedrine hydrochloride (—-),10 μg/mL of theophylline (….) and a mixture containing 10 μg/mL of HYX, 15 μg/mL of EPH, and 10 μg/mL of THP (-.-.-.) using methanol as a solvent.

**Fig 2** shows that the absorption spectra of HYX, EPH, and THP overlap significantly. Therefore, it is not possible to directly analyze the absorbances in the zero-order bands to detect any of these components, except for THP. THP can be calculated at its λ_max_ of 272 nm, free from the influence of EPH and HYX.

In this manuscript, The newly developed ratio-subtraction combined with derivative spectrophotometric method utilizes ratio subtraction and ^3^D derivative spectrophotometric methods to determine the triple combination with overlapped spectra. To develop the proposed method, different variables were studied and optimized.

***i) solvent effect***: during the study, different solvents such as methanol, ethanol, 0.1 N NaOH, 0.1 N HCl, and water were tested to determine their effect on the separation of the components. Among the tested solvents, methanol showed the highest sensitivity and satisfactory differentiated spectra for the components under study.

***ii) divisor concentration***: trials were conducted with divisor concentrations of 5, 10, and 22 μg/mL. The best accuracy and precision were obtained with a 22 μg/mL THP concentration as the divisor.

***iii) Derivative-spectrophotometry parameters*:** after experimenting with various choices of Δλ and scaling factor, good results of the ^3^D spectra produced with Δλ = 4 and scaling factor = 100.

The absorbance value of THP at its λ_max_ of 271 nm was correlated with the associated concentration interval of 2–24 μg/mL (**Fig 2**) to create a calibration curve for THP determination. The regression formula was revealed to be:

A_THP_ = 0.0467C_THP_ + 0.0061 r = 0.9998

where A is the absorbance value at 271 nm. C_THP_ is the concentrations of THP in μg/mL, and r is the correlation coefficient.

**Fig 3:**
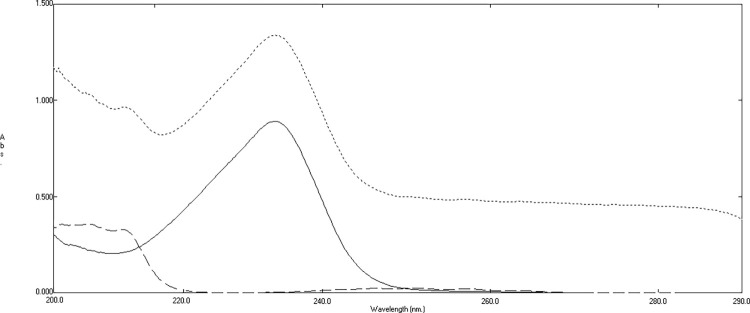
Division spectra of 10 μg/mL of hydroxyzine hydrochloride (―), 15 μg/mL of ephedrine hydrochloride (—-) and mixture containing 10 μg/mL of HYX, 15 μg/mL of EPH, and 10 μg/mL of THP (…..) using standard spectrum of 22 μg/mL theophylline as the divisor.

**Fig 4:**
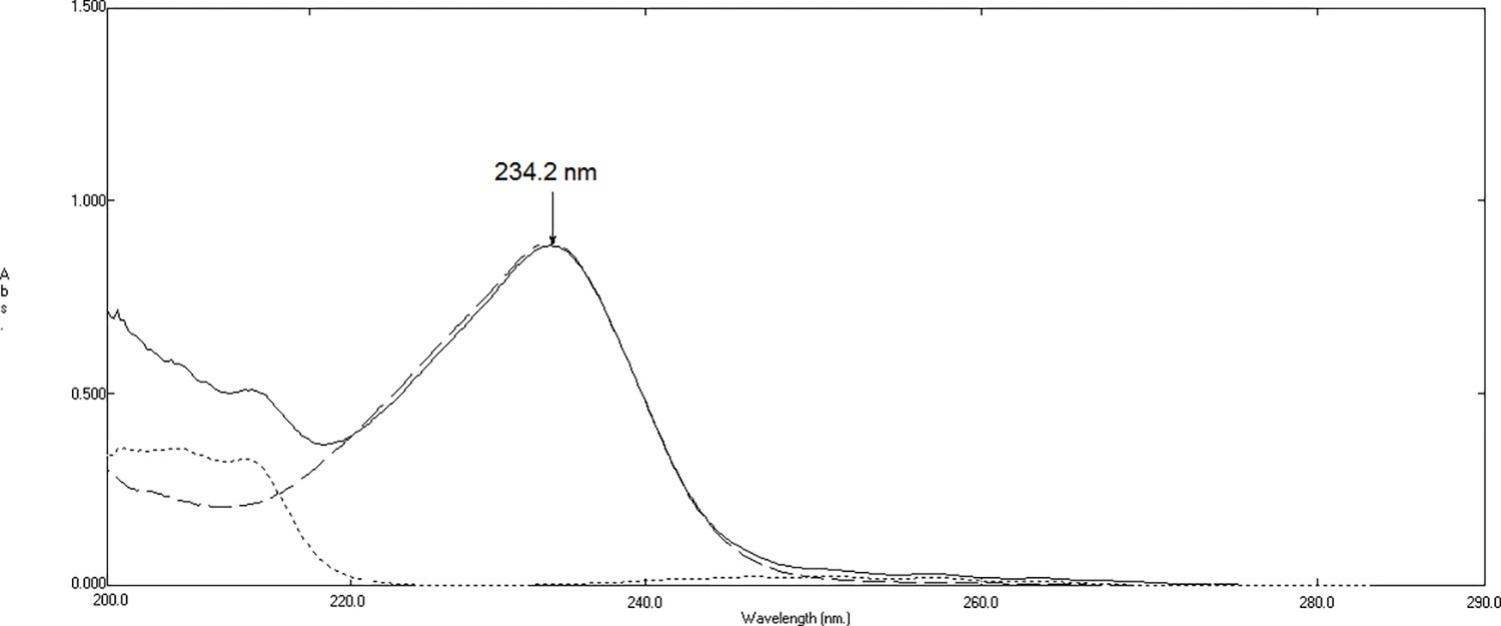
Division spectra of 10 μg/mL of hydroxyzine hydrochloride (―), 15 μg/mL of ephedrine hydrochloride overlaid with subtracted division spectra of mixture containing 10 μg/mL of HYX, 15 μg/mL of EPH, and 10 μg/mL of THP (…..).

To conclude the concentration of HYX, various concentrations of HYX in the range of 2–20 μg/mL were divided by the standard spectrum of 22 μg/mL THP. Then, the amplitude value at 271 nm in the plateau region was subtracted in the divided spectra. The resulting subtracted spectra were analyzed by calculating the peak amplitude at 234.2 nm, as disclosed in **Figs 3 and 4**. Based on the peak amplitude values, a calibration curve was generated, and a regression equation was established:

P.A_HYX_ = 0.0958 C_HYX_—0.0534 r = 0.9997 where P.A is the peak amplitude at 234.2 nm. C_HYX_ is the concentrations of HYX in μg/mL, and r is the correlation coefficient.

**Fig 5:**
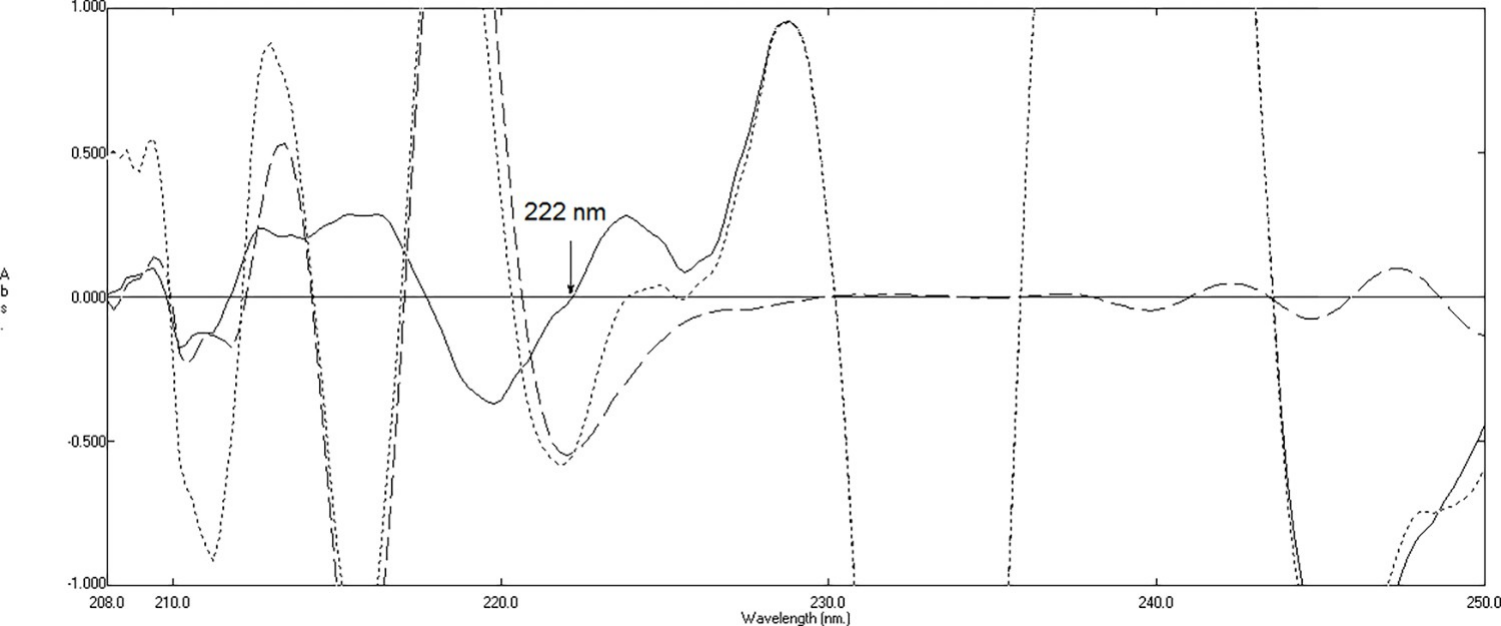
Third derivative absorption spectra of division spectra of 10 μg/mL of hydroxyzine hydrochloride (―), division spectra of 15 μg/mL of ephedrine hydrochloride (—-) and subtracted division spectra of mixture containing 10 μg/mL of HYX, 15 μg/mL of EPH, and 10 μg/mL of THP (…..).

To determine the EPH concentration, different concentrations ranging from 3 to 35 μg/mL were divided by the standard spectrum of THP, which was 22 μg/mL. The resulting ^3^D spectra were then processed using a Δλ value of 4 and a scaling factor of 100. Linear correlations between the peak amplitudes at 222 nm were then established for the ^3^D spectra of EPH, as displayed in **Fig 5**. The regression formula was computed as:

P.A_EPH_ = 0.0313 C_EPH_ + 0.0888 r = 0.9997

where P.A is the peak amplitude at 222 nm. C_EPH_ is the concentrations of EPH in μg/mL, and r is the correlation coefficient.

The results presented in **Table 2** demonstrate the accuracy, specificity, and validity of the proposed ratio-subtraction combining derivative spectrophotometric method for quantifying HYX, EPH, and THP in various laboratory-prepared mixtures.

### 4.2. Multivariate calibration techniques

Multivariate calibration is currently considered the most effective method for analyzing highly complex mixtures [51,52]. Multivariate calibration has the advantage of quickly identifying constituents without the need for initial separation. In comparison, traditional univariate analysis uses separate calibration curves for each constituent, which may not be accurate for mixtures with interferences and overlapping spectra. Multivariate calibration addresses this by simultaneously examining all the spectrum information [53].

We utilized multivariate calibration techniques such as PCR and PLS to calculate HYX, EPH, and THP in this procedure. Instead of relying on just one spectral wavelength, we included several wavelengths, which greatly increased our analysis’s accuracy and prediction power. These multivariate models have significantly improved spectrum analysis.

In multivariate calibration methods, building a calibration matrix is the initial stage toward concurrently determining the components in a triple mixture. The absorption spectra of 25 distinct combinations comprising HYX, EPH, and THP in various component ratios are utilized to create this matrix. The precise ratios of each component employed in the mixtures are shown in **Table 1**.

Excluding the spectral band above 210 nm and below 230 nm improved the study’s results. It was shown that the analysis was more accurate when this wavelength band was excluded. We used the ’leave one out’ cross-validation technique to evaluate the efficiency of the generated models. Using the samples that remain after deliberately deleting a single sample from the calibration set, a model that forecasts the concentration of the deleted sample is built. This process can be repeated for each sample in the calibration set to assess the model’s validity.

Two techniques, PCR and PLS, were utilized to develop various models. To demonstrate the predictive ability of these models, the Root Mean Square Error of Cross-Validation (RMSECV) values were calculated. Lower RMSECV values indicate better forecasting ability. By considering five parameters, it was determined that both PLS and PCR models produced satisfactory results. These factors, which represent the greatest variation in the dataset, are the principal components or latent variables taken from the spectral data.

To confirm the accuracy of the proposed models, an extra validation set (**Table 3**) was employed. The models were developed to forecast the HYX, EPH, and THP levels in this validation group. The acquired data presented in **Table 4** demonstrated a reasonable level of prediction accuracy.

**Table 3:** Results of determination of hydroxyzine hydrochloride, ephedrine hydrochloride and theophylline in laboratory prepared mixtures in the validation set using the proposed multivariate method.

Mix NO.	Ratio			Recovery %					
				PLS			PCR		
	HYX	EPH	THP	HYX	EPH	THP	HYX	EPH	THP
1	8	9	18	100.37	97.33	101.22	100.25	98.11	101.00
2	6	15	18	99.83	101.07	99.94	99.67	101.67	99.89
3	6	12	14	100.33	101.17	99.86	100.50	100.33	99.21
4	2	6	10	102.50	101.67	99.30	101.50	102.00	100.60
5	2	3	18	103.00	99.00	101.67	102.00	103.00	99.50
6	4	6	14	99.00	99.33	100.86	98.25	99.67	100.78
7	4	12	18	98.25	98.75	101.67	98.75	98.00	100.05
**Mean ±SD**				100.13 ±1.365	100.40 ±1.935	100.15 ±1.582	100.40 ±1.743	99.76 ±1.582	100.64 ±0.949

**Table 4:** Results of assay validation parameters of the proposed multivariate method for determination of hydroxyzine hydrochloride, ephedrine hydrochloride and theophylline.

Validation parameters	HYX		EPH		THP	
	PLS	PCR	PLS	PCR	PLS	PCR
**Mean ± SD**	100.13± 1.365	100.40± 1.743	100.40± 1.935	99.76± 1.582	100.15± 0.671	100.64± 0.949
**RMSEP**	0.0382	0.0424	0.1262	0.1317	0.0836	0.1399
**Predicted versus actual concentration plot** a-Slope b-Intercept c-Correlation coefficient (r)	0.9990 0.0015 0.9998	0.9965 0.0215 0.9998	1.0083 -0.0479 0.9995	1.0086 -0.0861 0.9994	0.9973 0.0623 0.9994	1.0281 -0.3469 0.9996

The validation sets’ estimated concentrations were compared to the actual concentration values to determine whether the model explained the concentration shifts. Each plot displayed an intercept close to zero and a slope close to one. Another diagnostic method used to identify errors in expected concentrations was the root mean square error of prediction (RMSEP), which measures both accuracy and precision [34]. The assay validation parameter results for the suggested models are presented in **Table 4**.

**Table 5** displays the results of the evaluation of the suggested ratio-subtraction combined with derivative spectrophotometric method’s accuracy, repeatability, and intermediate precision in accordance with ICH requirements. The suggested procedures were effectively used to determine the quantity of HYX, EPH, and THP in their pharmaceutical formulation (Bronchaline^®^ tablets). As seen in **Table 6**, the standard addition approach was used to determine that there was negligible interaction from the pharmaceutical formulation’s excipients.

**Table 5:** Results of assay validation parameters of the proposed ratio-subtraction derivative spectrophotometric method for determination of hydroxyzine hydrochloride, ephedrine hydrochloride and theophylline.

Parameters	HYX	EPH	THP
**Range (μg/mL)**	2–20	3–35	2–24
**Linearity** **Slope** **Intercept** **Correlation coefficient (r)**	0.0958 -0.0534 0.9997	0.0313 0.0888 0.9997	0.0467 0.0061 0.9998
**Accuracy (mean ± SD)**	100.02 ± 1.193	99.97± 1.255	99.97 ± 0.910
**Selectivity**	101.16 ± 1.408	100.96 ± 1.857	101.47 ± 1.257
**Precision (RSD%)** **Repeatability** ^**a**^ **Intermediate precision** ^**a**^	0.925 1.102	1.08 1.32	0.776 0.954

^**a**^ The intraday precision (n = 3), average of three different concentrations repeated three times within day. The interday precision (n = 3), average of three different concentrations repeated three times in three successive days.

**Table 6:** Statistical comparison of the results obtained by the proposed methods and the reported method for determination of hydroxyzine hydrochloride, ephedrine hydrochloride and theophylline in their pharmaceutical formulations.

Items	Bronchaline^®^ tablets									Reported method ^a^		
	Ratio-spectra combined with derivative spectrophotometry			Multivariate Calibration Techniques								
	HYX	EPH	THP	HYX		EPH		THP		HYX	EPH	THP
				PLS	PCR	PLS	PCR	PLS	PCR			
Mean	98.00	96.00	103.83	97.00 ^**b**^	96.00 ^**b**^	95.33 ^**b**^	94.00 ^**b**^	104.25 ^**b**^	105.08 ^**b**^	97.32	96.17	104.58
SD	1.489	1.282	1.335	1.328	1.126	1.552	1.301	1.093	1.109	1.222	1.472	1.213
%RSD	1.519	1.335	1.286	1.369	1.173	1.628	1.384	1.048	1.055	1.256	1.531	1.160
N	6	6	6	6	6	6	6	6	6	6	6	6
Variance	2.217	1.643	1.782	1.763	1.268	2.409	1.693	1.195	1.229	1.493	2.167	1.471
Standard addition technique (Mean ± SD) ^**c**^	100.67 ± 1.756	100.10 ±1.641	100.92 ± 1.283	101.49 ±1.510	99.80 ±1.587	100.94 ±1.419	100.68 ±1.769	100.07 ±1.405	101.12 ±1.694	—		
Student’s t-test (2.228) ^**d**^	1.582	0.178	0.901	0.348	0.869	0.715	0.987	0.267	0.966	—		
F-value (5.050) ^**d**^	1.503	1.319	1.210	1.180	1.161	1.112	1.279	1.230	1.197	—		

^**a**^ RP-HPLC method using a C18 column and mobile phase consisted of equal volumes of acetonitrile and 0.1% (w/v) aqueous ammonium carbonate buffer solution adjusted to pH 7 with acetic acid at a flow rate of 2 mL/min.

^**b**^ The ratio in Bronchaline^®^ tablets prepared by spiking technique where 4μg/mL of HYX is (3 μg/mL of pure drug + 1 μg/mL of pharmaceutical formulation), 6 μg/mL of EPH is (4.5 μg/mL of pure drug + 1.5 μg/mL of pharmaceutical formulation) and 12 μg/mL of THP.

^**c**^ Average of 3 determinations.

^**d**^ Figures between parenthesis represent the corresponding tabulated values of t and F at *P* = 0.05.

**Table 6** provides an analysis of variance to determine HYX, EPH, and THP in their pharmaceutical formulation. The results from the recommended methods were compared to those obtained from the reported HPLC approach [41] using student’s t and F-ratio tests. The estimated t- and F-values were found to be lower, indicating that there is no crucial difference.

### 4.3. The greenness score and applicability of the methods

The use of environmentally friendly chemicals and analytical techniques is crucial in today’s world to reduce the harmful impact of chemical reactions on the ecosystem and to improve the overall health of the planet. Green analytical assessment and green chemistry applications play a pivotal role in conserving resources, promoting economic growth and innovation, protecting the environment, ensuring human health, and achieving sustainable development goals. By utilizing these approaches, chemists, analyzers, and researchers can contribute significantly to developing more resilient and long-lasting strategies to safeguard both human safety and the environment.

Analyzing and assessing the environmental effects of analytical activities in chemistry is carried out by employing the Green Analytical Procedure Index, or GAPI [54]. Analytical techniques can be assessed using the GAPI framework on many environmentally important criteria. These variables could be the quantity of waste generated, the amount of energy and solvent utilized in the process, and the possibility of any hazardous materials present. Every criterion in the index has a color-coded score, and the total GAPI rating displays how much the analytical process has affected the environment [55]. The environmental safety of both ratio-subtraction combining derivative and multivariate calibration methodologies is indicated by the seven green, five yellow, and three red sections of the GAPI tool’s rating of the recommended spectrophotometric methods (**S1 Table)**.

The Analytical Greenness (AGREE) tool evaluates how analytical methods used in chemical analysis affect the surroundings [56]. It stipulates a systematic approach to evaluate the eco-friendliness of analytical techniques by considering various factors, such as hazardous materials usage, energy consumption, waste generation, and overall resource efficiency. The aim of AGREE is to promote the development and adoption of greener and sustainable analytical methodologies. The proposed approaches achieved scores of 0.61 and 0.58 using the AGREE tool when used alongside the progressed ratio-subtraction combining derivative and multivariate calibration methods, respectively, indicating that the proposed approaches are green (**S1 Table)**.

### 4.4. The method’s blueness and practicality

To assess the methods’ applicability and practicality, the new Blue Applicability Grade Index (BAGI) tool was adapted [57]. The BAGI tool allow judgment of the analytical method based on ten attributes giving an index score and an asteroid-like pictogram. Herein, BAGI scores obtained for the progressed ratio-subtraction combining derivative and multivariate calibration methods were 77.5 and 75, respectively. These good scores attributed to embracing simple spectrophotometric technique permit determination of several samples per hour, no sample pretreatment or derivatization is required, and no hazardous chemicals utilized (**S1 Table**).

### 4.5. Comparison with the reported HPLC methods

By comparing the provided spectrophotometric methods with the reported HPLC methods [41,42], it is clear that the newly proposed spectrophotometric methods are simpler and more cost-effective than the existing HPLC methods. The latter requires expensive solvents and specialized instrumentation. Additionally, the developed methods were found to be more environmentally friendly than the previously published ones, as assessed using the GAPI and AGREE tools. Moreover, the practicality of the developed methods was found to be like the reported ones when utilizing the BAGI tool, as demonstrated in **S1 Table**.

## 5. Conclusions

The advised spectrophotometric techniques effectively analyze concentrations of hydroxyzine hydrochloride, ephedrine hydrochloride, and theophylline in their ternary mixture, in powder or medication form. These techniques have been specifically designed and optimized to precisely measure each component in the studied mixture. They afford reliable and precise concentration monitoring. Spectrophotometric techniques are recognized for being simple, easy to use, fast, and cost-effective for the identification of prescription drugs. These techniques offer a useful alternative to chromatographic methods, especially in the pharmaceutical industry where they ensure the consistency and quality of pharmaceuticals.

The developed methods’ parameters were optimized and validated as per ICH guidelines. The newly developed Ratio-subtraction combined with derivative spectrophotometric method is selective and easy to apply allowing resolving the overlapped spectra of the ternary mixture either alone or in their formulation. The use of multivariate calibration techniques (PLS and PCR) has resolved several issues with the analysis of mixtures containing multiple components. These methods eliminate the need for pretreatment procedures and enhance the efficiency and convenience of the process. Accurate determination of the concentrations of theophylline, hydroxyzine hydrochloride, and ephedrine hydrochloride is crucial to ensure safe, effective, and high-quality medications. These techniques provide the necessary information to achieve this goal, benefiting researchers, pharmacists, and manufacturers.

The proposed spectrophotometric techniques were evaluated for their environmental impact using the GAPI and AGREE tools. Both assessments demonstrated that the recommended methods are environmentally safe and do not negatively impact ecology.

Additionally, the BAGI score was used to confirm the feasibility and practicality of the proposed methods, demonstrating their ease of use and compliance with mainstream white analytical chemistry objectives. Ultimately, these methods adhere to ecologically appropriate guidelines, which promote sustainable analytical practices and lower the environmental effects of analytical operations.

## Supporting information

S1 Table Greenness assessment of the proposed spectrophotometric methods and the reported methods using GAPI, AGREE, and BAGI approaches. (PDF)
