# Supplementary material for: Harnessing spectrophotometry resolution power for determining ternary mixture for respiratory disorders treatment in their pharmaceutical formulation
Source: PLoS One. 2024 Oct 7;19(10):e0311121. doi: 10.1371/journal.pone.0311121 (PMC11458049; doi:10.1371/journal.pone.0311121)
Supplement: S1 Table — (PDF) [file pone.0311121.s001.pdf]

S1 Table. Greenness assessment of the proposed spectrophotometric methods and the reported methods using GAPI, AGREE, and BAGI approaches.

Assessment tool	Ratio-spectra combined with derivative spectrophotometry	Multivariate Calibration Techniques	Reported HPLC method I [41]	Reported HPLC method II [42]
**GAPI**	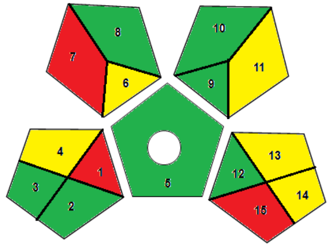	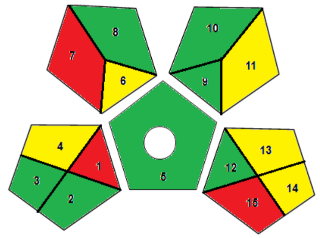	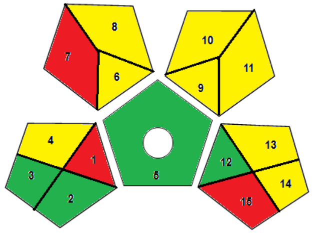	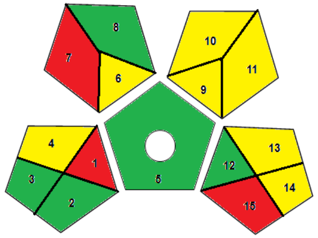
**AGREE**	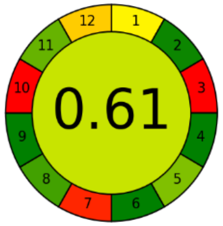	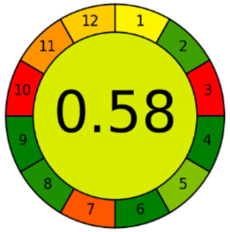	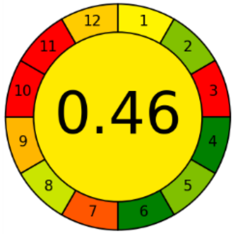	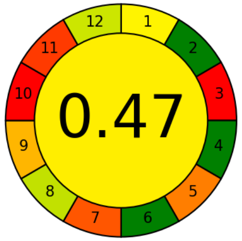
**BAGI**	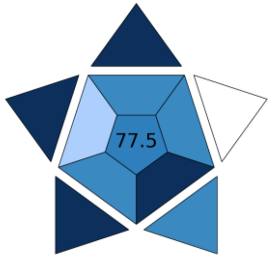	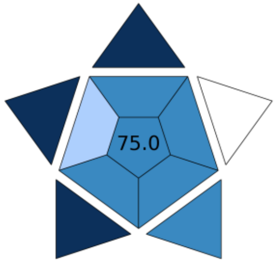	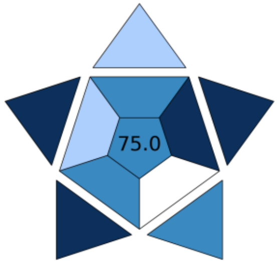	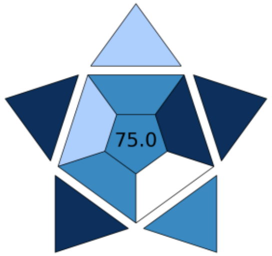
